# The impact of glucagon-like peptide-1 receptor agonists on objective physical activity in adults: a systematic review and exploratory meta-analysis

**DOI:** 10.1038/s41366-026-02141-z

**Published:** 2026-07-03

**Authors:** Heeyeon Kim, Ji Heon Chae, Hyo Youl Moon

**Affiliations:** 1 Department of Physical Education, Seoul National University, Gwanak-gu Seoul, Republic of Korea; 2 Institute of Sport Science, Seoul National University, Gwanak-gu Seoul, Republic of Korea

**Keywords:** Obesity, Obesity, Weight management, Homeostasis

## Abstract

Glucagon-like peptide-1 receptor agonists (GLP-1 RAs) are effective pharmacological treatments for obesity, through appetite suppression and weight reduction. However, their potential influence on physical activity (PA), a key determinant of long-term energy balance, remains unclear. This review aimed to evaluate whether GLP-1 RA treatment affects objectively measured PA in adults with overweight or obesity. A systematic review and exploratory meta-analysis were conducted in accordance with PRISMA guidelines. PubMed and Embase were searched from inception to March 10, 2026. Eligible studies included randomized and non-randomized controlled trials examining objectively measured PA outcomes (e.g., step counts, sedentary time, and intensity-specific PA). Narrative synthesis was performed across all studies, and a random-effects meta-analysis was conducted for studies reporting comparable free-living PA outcomes. Seven studies from six independent trials (*n* = 924) were included. Most studies reported no statistically significant differences in PA between GLP-1 RA and control groups. However, five studies (71.4%) showed numerically lower free-living PA in GLP-1 RA-treated groups, with one study reporting a significant reduction in daily step counts (–1144 steps/day, *p* = 0.01). Structured exercise-related outcomes, including adherence and prescribed exercise duration, were comparable between groups. Meta-analysis of three RCTs showed no significant pooled effect (Hedges’ g = –0.11, 95% CI –0.30 to 0.09, I^2^ = 0.0%). Sensitivity analyses suggested a modest reduction in PA (Hedges’ g = –0.24, 95% CI –0.46 to –0.02, *p* = 0.035, I^2^ = 34.3%), influenced by study design and outcome heterogeneity. GLP-1 RA treatment does not significantly alter overall PA but may be associated with a small reduction in free-living PA, while structured exercise participation remains unaffected. These findings suggest a potential influence on spontaneous, non-obligatory activity rather than exercise capacity. Further studies using standardized objective PA measures are needed.

## Introduction

Considering the rising prevalence of obesity worldwide, researchers have made continuous efforts to develop effective weight loss strategies to mitigate its complications and improve well-being [1, 2]. Glucagon-like peptide-1 receptor agonists (GLP-1 RAs), initially developed for the treatment of type 2 diabetes mellitus (T2DM), have recently emerged as effective therapeutics for obesity [3, 4]. Exenatide was the first short-acting GLP-1 RA approved for weight management by enhancing satiety [5]. Subsequently, long-acting GLP-1 RAs such as liraglutide [6], semaglutide [7], and tirzepatide [8] demonstrated substantial efficacy in weight reduction.

GLP-1 is an incretin peptide hormone secreted by enteroendocrine L cells in the small intestine in response to food intake [9]. It binds to GLP-1 receptors expressed in pancreatic beta cells, the central nervous system, hindbrain, forebrain and blood vessels [10]. Activation of GLP-1 receptors inhibits glucagon secretion, increases insulin sensitivity, delays gastric emptying, suppresses appetite, and enhances satiety signaling [9, 11]. GLP-1 RAs are synthetic analogs of human GLP-1 designed for prolonged stability by resisting degradation by dipeptidyl peptidase-4 (DPP-4) [12]. While GLP-1 RAs effectively reduce caloric intake and promote weight loss, their multi-organ effects necessitate consideration of potential adverse events [13]. Most reported adverse events are gastrointestinal [14], yet evidence regarding long-term physiological consequences remains limited.

Although GLP-1 RAs have demonstrated substantial efficacy in weight reduction, long-term weight management remains challenging, particularly in real-world settings where treatment discontinuation is common [15, 16]. Weight regain following cessation of pharmacological treatment is frequently observed and is thought to be driven primarily by metabolic adaptations, with potential contributions from behavioral factors [17, 18]. In this context, spontaneous physical activity, commonly conceptualized as non-exercise activity thermogenesis, represents a key behavioral component of long-term weight maintenance [19]. In addition to effects on appetite regulation, GLP-1 RAs have been shown to modulate neurocortical circuits involved in reward and motivation [20, 21]. Such alterations in brain activity may influence motivation and fatigue perception [22, 23], potentially affecting engagement in PA [24]. However, whether these neurobehavioral effects translate into measurable changes in objectively measured PA in humans remains unclear.

Understanding the potential influence of GLP-1 RAs on PA patterns is clinically relevant, as changes in PA may modify overall energy balance and influence long-term weight management outcomes [18]. Therefore, this systematic review aimed to investigate whether GLP-1 RAs influence objectively measured PA in adults with overweight or obesity. The findings may provide insights into the physiological mechanisms underlying behavioral changes and underscore the need to refine lifestyle interventions to support sustainable weight management.

## Methods

### Search methodology

**Fig. 1:**
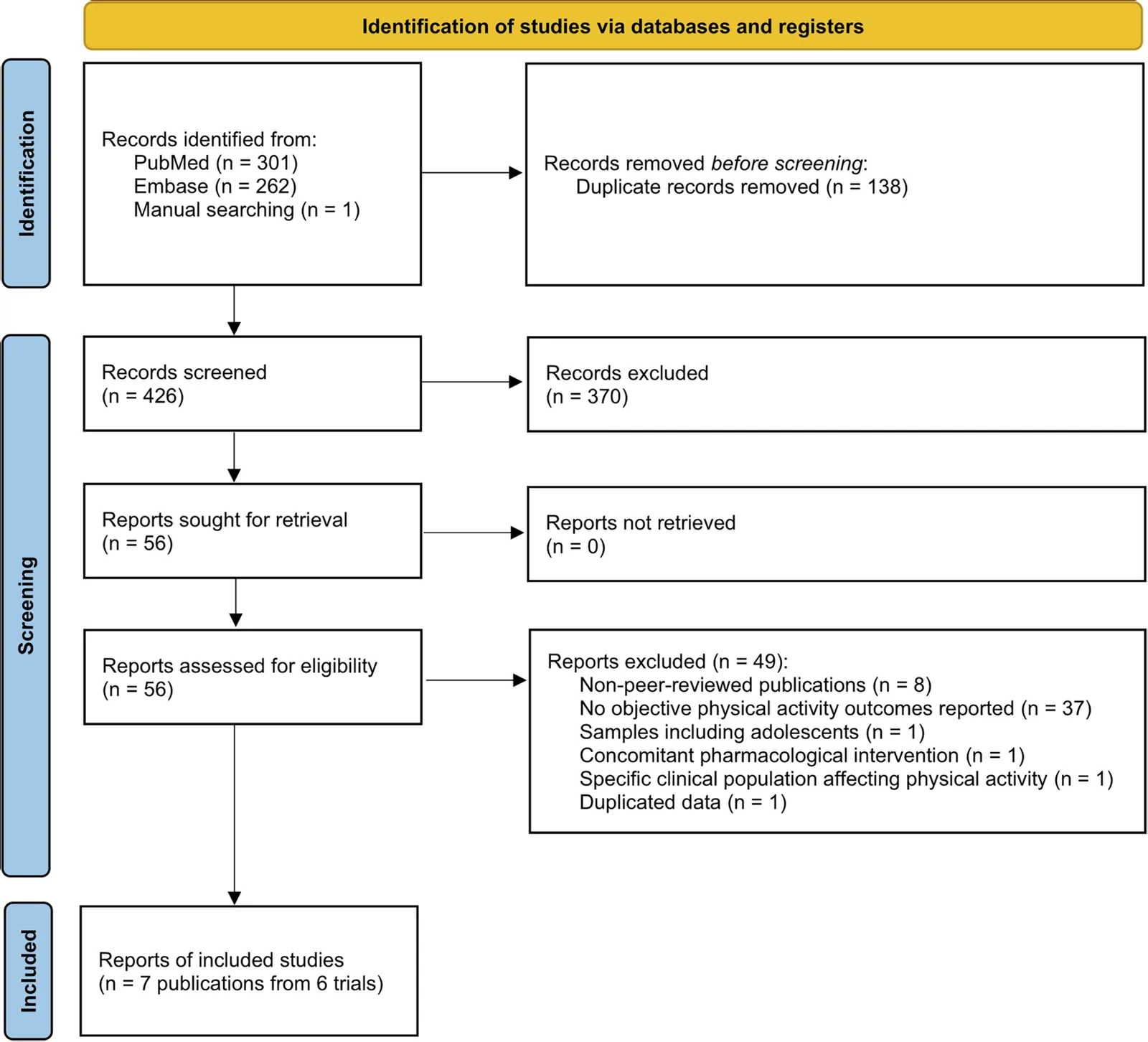
PRISMA 2020 flow diagram of study selection.

The systematic review and an exploratory meta-analysis were conducted in accordance with the Preferred Reporting Items for Systematic Reviews and Meta-Analysis (PRISMA) guidelines (Fig. 1) [25]. The review protocol was pre-registered with the International Prospective Register of Systematic Reviews (PROSPERO ID CRD420251149477). Literature search was conducted across two electronic databases (PubMed and Embase) from inception to March 10, 2026. Controlled vocabulary terms (e.g., MeSH and Emtree) were used to ensure a comprehensive search. The search terms included, but were not limited to, ‘GLP-1 receptor agonist’, ‘physical activity’, ‘exercise’, ‘accelerometer’, which were combined using Boolean operators (OR and AND) (Supplementary Material 1).

### Inclusion and exclusion criteria

The inclusion criteria were established based on the PICO framework. Participants were adults (aged 18 years or older) receiving GLP-1 RAs for overweight or obesity management. Studies involving individuals with common obesity-related comorbidities, such as type 2 diabetes mellitus or osteoarthritis, were included, as these conditions are highly prevalent in the target population and reflect real-world clinical settings. To isolate the effect of GLP-1 RA treatment, studies were included if lifestyle interventions (e.g., diet and exercise programs) were applied equally across intervention and control groups or were appropriately controlled within the study design. Comparators included placebo, usual care, or other antidiabetic medications (e.g., sodium-glucose cotransporter 2 inhibitor, metformin, DPP-4 inhibitor). Outcomes of interest were real-world, objectively measured PA variables, including step count, sedentary time, and light- to vigorous-intensity PA. In studies that incorporated structured exercise interventions, objectively measured exercise participation was also included as a PA outcomes. When available, total daily energy expenditure derived from objective monitoring was also considered. Only experimental studies, including randomized controlled trials (RCTs), and non-randomized controlled trials, published in English were included. Secondary analyses of eligible trials were included when they reported relevant PA outcomes not presented in the primary publication.

Studies were excluded if they involved participants with type 1 diabetes mellitus or conditions that could directly influence PA independent of GLP-1 RA treatment (e.g., hypothalamic disorders, neurologic disorders). Studies with concomitant pharmacological interventions that could independently affect PA (e.g., testosterone replacement therapy) were excluded. Pediatric populations and animal studies were also excluded. Studies reporting outcomes unrelated to free-living PA or assessing exercise capacity solely under controlled laboratory conditions (e.g., 6-minute walking distance test, maximum oxygen consumption (VO_2_max) test) were excluded, as they reflect physiological capacity rather than real-world physical activity behavior. Observational studies, reviews, and non-peer-reviewed publications (e.g., editorials, conference abstracts, protocols, and preprints) were also excluded.

When multiple publications originated from the same study population, each was evaluated for outcome-specific eligibility. Studies reporting distinct PA outcomes or analytical perspectives were included, and data from the same participants were not treated as independent evidence within each outcome domain.

### Study selection

Retrieved records were exported to Microsoft Excel (version 2021, Microsoft Corporation, Redmond, WA, USA) to remove duplicates. Subsequently, an independent reviewer examined all titles and abstracts for eligibility, followed by a full-text review based on the predefined inclusion criteria. Controversial cases were reviewed by two authors and discrepancies were resolved through discussion with a third reviewer. The final selected studies were cross-checked and managed using Zotero (version 7.0, Corporation for Digital Scholarship, Vienna, VA, USA).

### Data extraction

Data extraction was conducted using Microsoft Excel by one reviewer and verified by two additional reviewers. Any discrepancies were resolved through discussion. Extracted data included: (1) study characteristics (first author, publication year, study design, and country); (2) participant characteristics (sample size, mean age, baseline body mass index (BMI), and bodyweight change); (3) medication details (type of GLP-1 RA, administration route, duration, frequency, and dose); (4) additional interventions (type, duration, and protocol); (5) objectively measured PA outcomes; (6) results related to PA. Outcomes such as cardiorespiratory fitness and muscular strength were not extracted as they reflect physiological capacity rather than free-living physical activity behavior.

For studies with multiple intervention arms, all groups were initially extracted for descriptive purposes. For analytical comparisons, only predefined contrasts were included to isolate the effect of GLP-1 RA treatment. Specifically, comparisons were restricted to (1) GLP-1 RA monotherapy versus corresponding control groups and (2) combined GLP-1 RA plus exercise intervention versus exercise-only groups, where applicable.

### Data analysis

Given the substantial clinical and methodological heterogeneity across the included studies, a formal meta-analysis across all studies was not performed. Instead, a two-pronged analytical approach was adopted: a narrative synthesis for all included studies and an exploratory meta-analysis for a subset of studies reporting sufficiently comparable PA outcomes.

When studies did not report exact *p*-values or effect sizes, approximate estimates were derived from available summary statistics where possible. Standard deviations (SD) were estimated from reported means and 95% confidence intervals (CI) using established methods described in the Cochrane Handbook [26]. In addition, approximate *p*-values and effect sizes (Cohen’s *d*) were calculated to describe the direction and magnitude of between-group differences [27, 28]. These estimates were used for descriptive purposes only and were not included in the exploratory meta-analysis. When necessary, estimated SDs were incorporated into the meta-analysis.

For the exploratory meta-analysis, effect sizes were calculated using Hedges’ g to enable standardization across heterogeneous PA measures and were derived using the most consistently reported outcomes. Negative values indicate lower PA in the GLP-1 RA group compared with the control. A random-effects model using the DerSimonian and Laird method [29] was applied to account for between-study heterogeneity. The exploratory meta-analysis was conducted using R (R Foundation for Statistical Computing, Vienna, Austria). Results are presented as a forest plot. Given the limited number of included studies and heterogeneity, a full meta-analysis was not appropriate; therefore, an exploratory meta-analysis was conducted to provide hypothesis-generating insights.

### Risk of bias assessment

The risk of bias (RoB) was assessed using Cochrane Risk of Bias tool version 2.0 (RoB-2) and Risk Of Bias In Non-randomized Studies of Interventions (ROBINS-I) tool [30, 31]. Two researchers independently conducted the assessments, and disagreements were resolved through consensus. Results were visualized using the ‘robvis’ web application [32]. For secondary analyses or pooled analyses, RoB was assessed based on the methodological quality of the original contributing trials. When pooled data from multiple trials were reported, the risk of bias was evaluated across all contributing studies. When multiple publications originated from the same trial, they were assessed as a single study.

## Results

### Study selection

A total of 563 records were retrieved from two electronic databases (Fig. 1). Additionally, one record was identified through manual reference searching, yielding a total of 564 records in the identification phase. After removing 138 duplicates, 426 records remained for the title and abstract screening. Among the 56 articles assessed for full-text review, 49 were excluded due to being non-peer-reviewed publications (*n* = 8), lack of objectively measured PA outcomes (*n* = 37), inclusion of adolescent samples (*n* = 1), concomitant pharmacological intervention (e.g., testosterone replacement therapy) (*n* = 1), specific clinical populations affecting PA (e.g., craniopharyngioma) (*n* = 1), and duplicated data from previously selected studies (*n* = 1). Therefore, seven studies derived from six independent clinical trials were included in the systematic review [33–39]. Two publications [36, 37] originated from the same clinical trial (NCT04122716) and included multiple intervention arms. For the review, only relevant arms were selectively extracted: Jensen et al. [36] contributed data from non-exercise comparisons (GLP-1 RA monotherapy versus control), whereas Lundgren et al. [37] contributed data from exercise-related comparisons (GLP-1 RA plus exercise versus exercise alone). These were treated as independent comparisons, as no overlapping participant data were included in the same analysis.

### Study characteristics

Table 1 summarizes the characteristics of the included studies. Publication years ranged from 2010 to 2022, and all trials were conducted in Western countries. A total of 924 participants were included, with mean ages ranging from 40 to 58 years. All participants were classified as having overweight or obesity, with or without chronic diseases (e.g., diabetes mellitus, osteoarthritis). Three studies [34, 36, 37] investigated GLP-1 RA as an adjunct to weight-loss maintenance following an initial diet-induced weight reduction of at least 5% from baseline. The remaining studies initiated GLP-1 RA during active weight-loss. This distinction was considered when interpreting PA outcomes, as behavioral and metabolic adaptations may differ between maintenance and weight-loss phases.

**Table 1:** Baseline characteristics of the included studies.

First author (Year)	Country	Study Group description	Mean age (years)	Baseline BMI (kg·m^−2^)	Sample size (*n*)		Mean BW change (kg)	
					GLP-1 RA	Con	GLP-1 RA	Con
Apovian (2010)[33]	USA	Overweight to moderate obesity with T2DM	54.8 ± 9.5	33.8 ± 4.0	96	98	–6.2	–4.0
Bartholdy (2022) [34]^a^	Denmark	Obesity with knee osteoarthritis	58.7 ± 10.4	32.2 ± 5.1	66	69	–6.4	–2.3
Grannell (2021) [35]	Ireland	Severe obesity	54.4 ± 10.7	43.0 ± 6.1	59	19	–12.2	–9.7
Jensen (2022) [36]^ab^	Denmark	Obesity without DM	45.0 ± 12.0	32.4 ± 2.9	36	39	–1.9	+6.1
Lundgren (2021) [37]^ab^	Denmark	Obesity without DM	43.0 ± 12.0	32.6 ± 2.9	49	48	–3.4	+2.0
Tronieri (2020) [38]	USA	Obesity without DM	47.2 ± 11.5	39.0 ± 7.0	142	140	–8.1	–4.3
Yates (2022) [39]^c^	UK	Obesity with T2DM	52.0 ± 7.4^d^	33.8 ± 6.4^d^	20	43	–4.1	–1.4

Values are presented as mean ± SD where available.

*BMI* body mass index, *BW* body weight, *Con* control group, *GLP-1 RA* glucagon-like peptide-1 receptor agonist, *SD* standard deviation, *T2DM* type 2 diabetes mellitus.

^a^Baseline BMI and body weight values for Bartholdy et al., Lundgren et al. and Jensen et al. were measured after an initial low-calorie diet-induced weight loss phase. Reported body weight changes therefore reflect changes during the weight maintenance phase rather than initial weight loss.

^b^Only intervention arms corresponding to the predefined analytical comparisons are presented.

^c^In the pooled analysis by Yates et al., GLP-1 RA and control groups were derived from separate trials and were not directly randomized within a single study.

^d^Estimated standard deviation derived from reported 95% confidence intervals and sample sizes.

A total of 468 participants received GLP-1 RA treatment, with mean body weight change ranging from –1.9 to –12.2 kg. Although Jensen et al. [36] conducted an exploratory analysis using a subset of participants from the same trials as Lundgren et al. [37], no double-counting occurred, as non-overlapping intervention arms were selectively extracted. Yates et al. [39] reported PA outcomes from a pooled analysis of three RCTs [40–42]. The GLP-1 RA group was derived exclusively from one (LYDIA; *n* = 20) [42], whereas the pooled usual care control group comprised participants from two separate trials (DIASTOLIC and SEESAW; *n* = 43) [40, 41]. This imbalance in trial contribution was considered when interpreting the findings, as between-trial heterogeneity may limit direct comparability. Accordingly, this study was included in sensitivity analyses and interpreted with caution.

### Risk of bias assessment

**Fig. 2:**
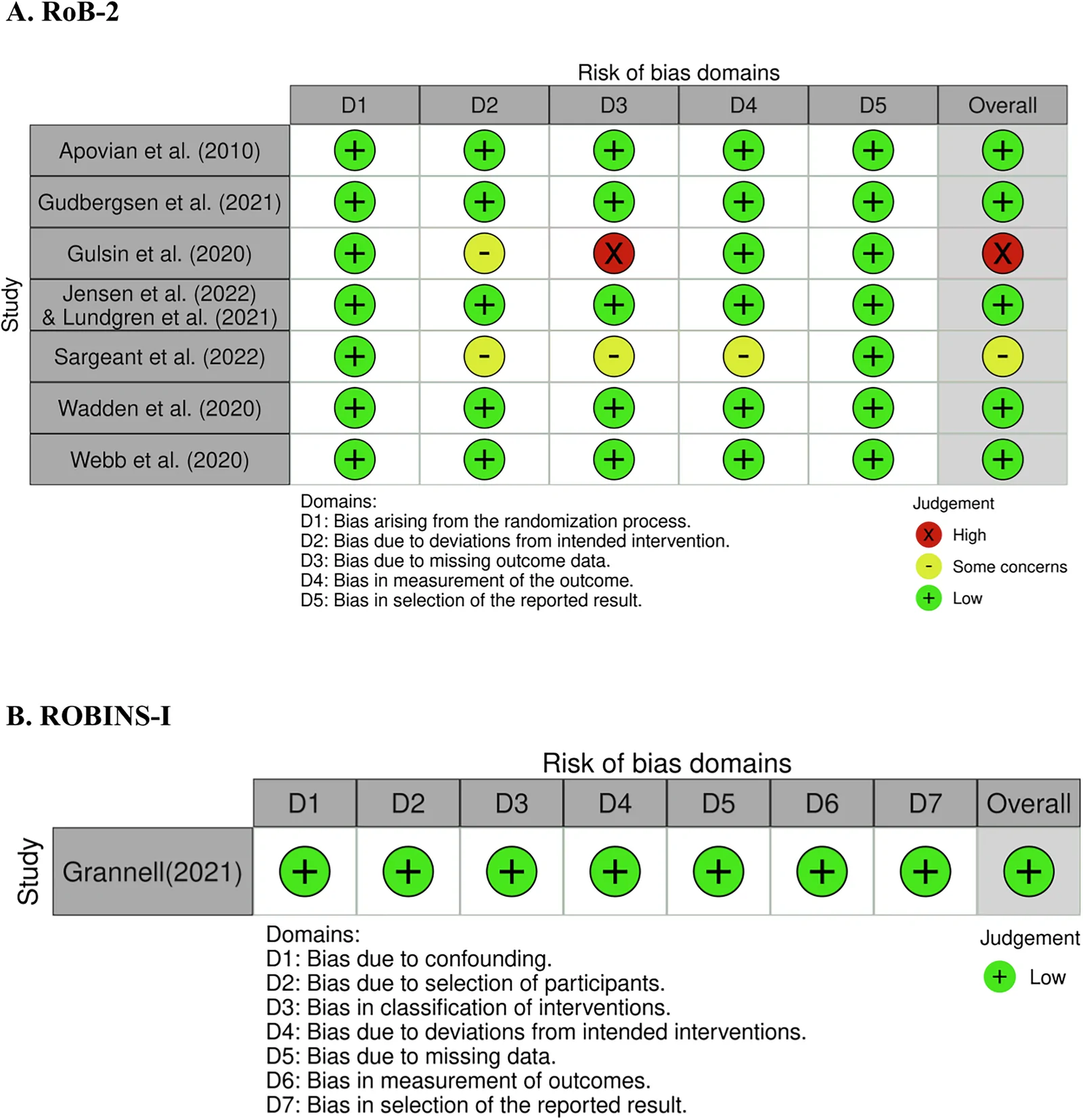
Risk of bias assessment of the included studies [33, 35–37, 40–42, 61, 62]. **A** Risk of bias assessment of randomized controlled trials using the Cochrane RoB-2 tool. **B** Risk of bias assessment of the non-randomized study using the ROBINS-I tool. Among the randomized trials assessed with RoB-2, most were rated as low risk of bias, with some concerns in selected domains, and one study (Gulsin et al.) was rated as having a high overall risk of bias. The non-randomized study (Grannell et al.) was judged to have a low risk of bias across all domains.

The results of the risk of bias assessment are presented in Fig. 2. One study [35] was evaluated using ROBINS-I and judged to have an overall low risk of bias across all domains. Among the studies assessed with RoB-2, four (66.7%) were rated as low risk of bias, one (16.7%) as some concerns, and one as high risk of bias, primarily due to insufficient reporting on missing outcome data. Most concerns were related to missing outcome data and deviations from intended interventions, whereas randomization and outcome measurement were generally well reported. The high risk-of-bias rating for Gulsin et al. [40] (DIASTOLIC trial) was primarily driven by incomplete reporting on accelerometer-based PA data handling. Importantly, this study did not contribute to the GLP-1 RA effect estimates, and therefore does not bias the direction of treatment effect but may increase variability in the pooled usual care control group within Yates et al. [39]. The GLP-1 RA arm in Yates et al. was derived from Webb et al. [42] (LYDIA trial), which was rated as low risk of bias. Accordingly, the high risk of bias in Gulsin et al. mainly affects the control arm within the Yates comparison, and its influence should be considered when interpreting between-group differences. However, the GLP-1 RA effect estimates were largely informed by studies assessed as having low risk of bias.

### Lifestyle interventions

During the GLP-1 RA treatment, lifestyle interventions varied across studies, ranging from structured exercise programs to general lifestyle advice, which may contribute to heterogeneity in PA outcomes. Five studies (71.4%) provided exercise advisement, and six studies (85.7%) included dietary instruction supervised by specialists (Table 2). No structured lifestyle interventions were implemented in the trials contributing to the pooled data analysis by Yates et al. [39]. Dietary strategies typically involved calorie restriction (e.g., ~600 kcal/day deficit or 1200–1800 kcal/day intake), with one study implementing a high-protein diet (1 g/kg bodyweight) [35]. Exercise interventions varied, including supervised cycling sessions or self-directed aerobic activities, generally aligned with WHO recommendations (≥150 min/week of moderate-intensity PA) [43].

**Table 2:** Lifestyle interventions provided alongside GLP-1 RA treatment.

First author (year)	Diet	Exercise
Apovian (2010) [33]	Balanced macronutrient-content, calorie-restricted diet (~600 kcal/day deficit) instructed by a registered dietitian	Instructed to increase moderate-intensity PA to a minimum of 150 min/week
Bartholdy (2022) [34]	Dietician-led 8-week intensive formula diet (800–1000 kcal/day) during run-in; followed by self-managed calorie restriction with dietitian support (~1500 kcal/day)	No structured exercise program; participants were encouraged to maintain usual activity levels
Grannell (2021) [35]	Calorie restriction with high-protein, low GI diet (male 1500 kcal/day; female 1200 kcal/day; protein 1 g/kg BW), using two meal replacements per day plus one self-selected meal	Progressive RT advised 3 days/week (machine-based; 3 sets × 10–12 reps at RPE 6–7/10) + AT of moderate-to-high intensity ≥150 min/week; managed by an exercise physiologist
Jensen (2022) [36]^a^	Dietary consultation based on Danish national guidelines with moderate caloric restriction and emphasis on high satiety foods	Vigorous-intensity supervised group cycling (30 min) + circuit training (15 min) 2 days/week; moderate-to-vigorous intensity individual AT 2 days/week^b^
Lundgren (2021) [37]^a^	Same as Jensen et al. [36]	Same as Jensen et al. [36]
Tronieri (2020) [38]	BW-based calorie restriction (1200–1800 kcal/day; 15–20% protein, 20–35% fat, remaining from carbohydrates); daily food and calorie intake self-monitored using paper or electronic records	Moderate-intensity aerobic PA prescribed at 100 min/week and progressively increased by 25 min every 4 weeks to a target of 250 min/week, spread over 4–5 days
Yates (2022) [39]^c^	*Ad libitum* diet; general lifestyle advice provided	No structured exercise program; general advice to become more active provided

*AT* aerobic training, *BW* body weight, *GI* glycemic index, *PA* physical activity, *RT* resistance training.

**ᵃ**Lifestyle intervention details for Lundgren et al. [37] were identical to those described in Jensen et al. [36], both derived from the same parent trial (NCT04122716).

**ᵇ**In Jensen et al. [36], only participants from the non-exercise arm (liraglutide vs. placebo) were included in the present review; accordingly, the exercise intervention described here was not applied to the extracted participants.

**ᶜ**Yates et al. [39] pooled data from three separate RCTs; none of the contributing trials implemented a structured dietary or exercise intervention, and general lifestyle advice was provided to all participants.

### GLP-1 RA treatment

Exenatide and liraglutide were used across the included studies (Table 3). Exenatide was administered twice daily (5 μg titrated to 10 μg), whereas liraglutide was administered once daily (titrated up to 3.0 mg or 1.8 mg depending on study). Control groups included volume-equivalent placebo, usual care, or no pharmacological treatment. Dosing regimens and treatment durations varied across studies, which should be considered when interpreting between-study differences.

**Table 3:** GLP-1 RA pharmacotherapy details and physical activity outcomes.

First author (year)	Design	Type of GLP-1 RA	Dose/duration/route	Frequency	Comparator	PA measures	Main findings
Apovian (2010) [33]	RCT	Exenatide	5 μg SC for 4 weeks, then 10 μg SC up to week 24	Twice daily	Volume-equivalent placebo (SC)	METs, exercise-derived energy expenditure (kcal/min)	METs and energy expenditure increased from baseline in the placebo group but not in the exenatide group; no significant between-group difference
Bartholdy (2022) [34]	Post-hoc analysis of RCT	Liraglutide	3 mg SC for 52 weeks	Once daily	Volume-equivalent placebo (SC)	Total PA time (min/day); SENS-MOTION® accelerometer	No significant between-group difference in total PA time; both groups showed similar small increases from baseline
Grannell (2021) [35]	Non-RCT	Liraglutide	3 mg SC for 16 weeks	Once daily	No pharmacotherapy	RT frequency (days/week) and AT time (min/week); Milon software	No significant between-group difference; numerically lower RT frequency and similar aerobic exercise volume in the liraglutide group
Jensen (2022)[36]	Secondary analysis of RCT	Liraglutide	3 mg SC for 52 weeks	Once daily	Volume-equivalent placebo (SC)	Sedentary time, light-intensity PA, MVPA (min/day); GENEActiv wrist-worn accelerometry	No significant between-group difference in any PA parameter; numerically lower light-intensity PA and higher sedentary time in the liraglutide group
Lundgren (2021) [37]	RCT	Liraglutide	3 mg SC for 52 weeks	Once daily	Volume-equivalent placebo (SC)	Weekly exercise duration (min/week) and adherence (%); Polar A300	No significant difference in exercise adherence or duration between the combination and exercise-only groups
Tronieri (2020) [38]	Post-hoc analysis of RCT	Liraglutide	3 mg SC for 56 weeks	Once daily	Volume-equivalent placebo (SC)	PA adherence (%); Polar® Loop2	No significant between-group difference in PA adherence; adherence declined progressively in both groups over 56 weeks
Yates (2022)[39]^a^	Pooled data analysis of RCTs	Liraglutide	1.8 mg SC for 12 weeks	Once daily	No treatment (usual care)	Daily step count, MVPA (min/day)^b^	Significant reduction in daily step count and MVPA with liraglutide vs. pooled usual care control

*AT* aerobic training, *GLP-1 RA* glucagon-like peptide-1 receptor agonist, *METs* metabolic equivalent tasks, *MVPA* moderate-to-vigorous intensity physical activity, *PA* physical activity, *RCT* randomized controlled trial, *RT* resistance training, *SC* subcutaneous.

**ᵃ**Yates et al. [39] pooled data from three separate RCTs; the liraglutide arm was drawn exclusively from the LYDIA trial (*n* = 20), while the pooled usual care control arm consisted of participants from the DIASTOLIC and SEESAW trials (*n* = 43).

**ᵇ**Yates et al. [39] analyzed pooled data with generalized estimating equations; change from baseline in measures of PA was investigated by treatment group, with adjustment for baseline characteristics.

### Physical activity

PA outcomes were assessed using device-based objective measures across all studies; however, the specific constructs varied between free-living PA (e.g., accelerometer-derived step counts, moderate-to-vigorous PA, sedentary time) [33–36, 39] and structured or prescribed exercise-related metrics (e.g., adherence, exercise duration) [37, 38] (Table 3).

Across the seven studies, five (71.4%) reported numerically lower PA in GLP-1 RA-treated groups compared with controls, although only one study (Yates et al. [39]) demonstrated a statistically significant reduction. Apovian et al. [33] reported no significant between-group differences in METs or exercise-derived energy expenditure, despite numerically lower values in the GLP-1 RA group (e.g., METs: 0.9 ± 0.6 vs 1.6 ± 0.6, exenatide vs placebo, *p* = 0.39). Bartholdy et al. [34], Grannell et al. [35], Jensen et al. [36], and Lundgren et al. [37] consistently reported no significant differences across multiple PA-related outcomes. Yates et al. [39] observed a significant reduction in daily step counts with liraglutide (–1144 steps/day; 95% CI –2069 to –220; *p* = 0.01). A similar direction of effect was observed for moderate-to-vigorous PA, with significant reductions reported; however, quantitative effect estimates were not explicitly provided.

Studies assessing structured exercise metrics (e.g., adherence and prescribed exercise duration), also derived from device-based monitoring, reported no significant between-group differences, with similar adherence trajectories over time (Lundgren et al. [37]; Tronieri et al. [38]).

**Fig. 3:**
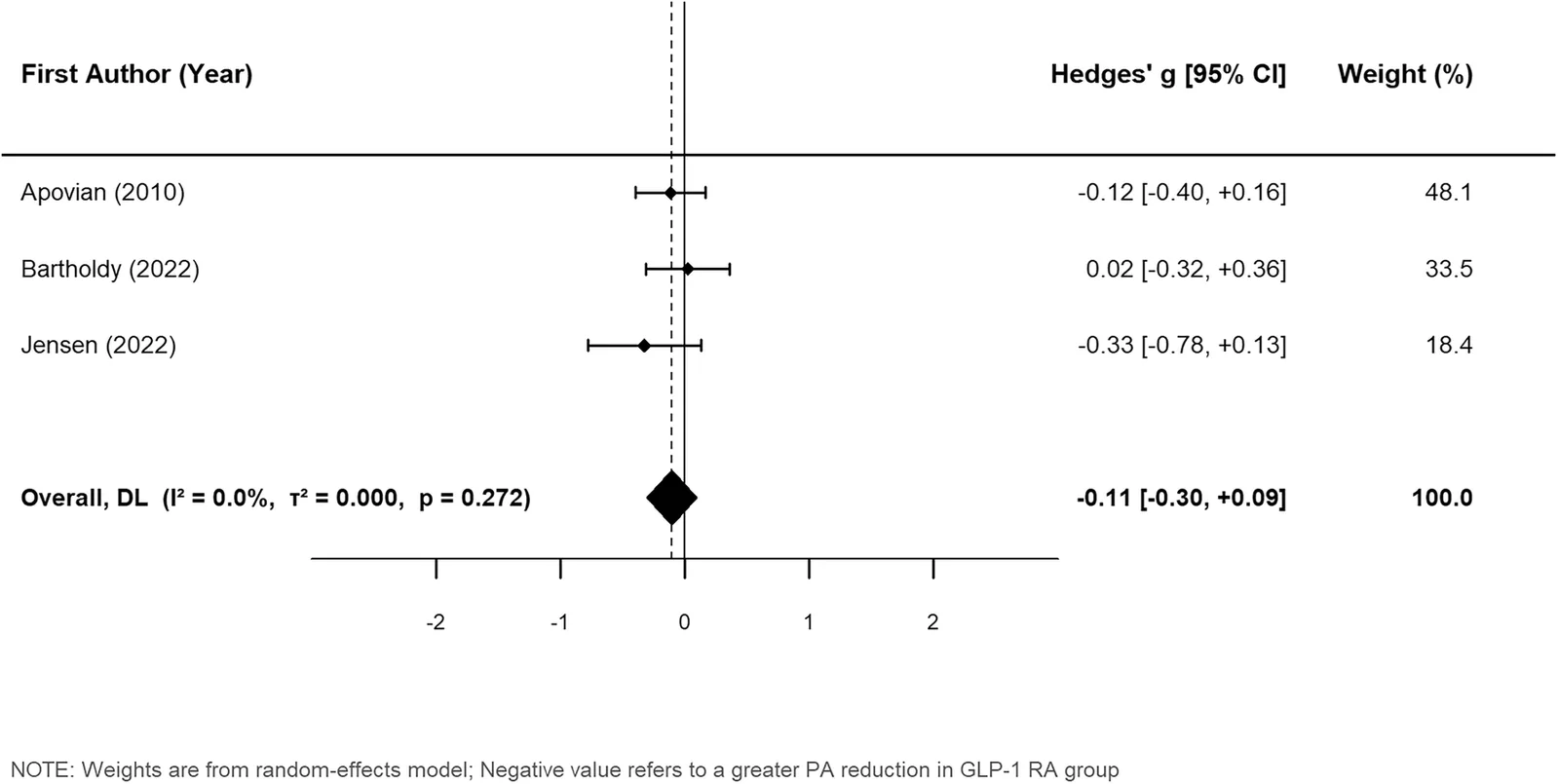
Forest plot of the exploratory meta-analysis (k = 3) examining the effect of GLP-1 receptor agonists (GLP-1 RAs) on objectively measured physical activity. Effect sizes are presented as Hedges’ g with 95% confidence intervals (CI) using a random-effects DerSimonian-Laird (DL) model. Negative values indicate a greater reduction in physical activity in the GLP-1 RA group compared with controls.

To quantitatively synthesize comparable outcomes, exploratory meta-analysis was restricted to free-living PA measures (Fig. 3). An exploratory meta-analysis including three RCTs (k = 3) [33, 34, 36] showed no significant difference between GLP-1 RA and control groups (Hedges’ g = –0.11, 95% CI –0.30 to 0.09) with no heterogeneity (I^2^ = 0.0%). Inclusion of pooled data from Yates et al. [39] (k = 4) did not materially change the results (Hedges’ g = –0.20, 95% CI –0.44 to 0.04), although heterogeneity increased to a low-to-moderate level (I^2^ = 37.6%) (Supplementary Material 2). Further inclusion of a non-randomized study (k = 5) [35] yielded a statistically significant pooled effect favoring reduced PA in the GLP-1 RA group (Hedges’ g = –0.24, 95% CI –0.46 to –0.02, *p* = 0.035), with moderate heterogeneity (I^2^ = 34.3%) (Supplementary Material 3).

Overall, while the primary analysis based on high-quality RCTs did not demonstrate a statistically significant effect, sensitivity analyses suggested that the observed effect may be influenced by study design, data structure, and outcome definition.

## Discussion

The present review examined whether GLP-1 RAs influence objectively measured PA in adults with overweight or obesity, with a focus on both free-living activity and structured exercise participation. Across the included studies, no statistically significant differences were observed in most PA outcomes. However, a consistent tendency toward lower free-living PA was observed in GLP-1 RA-treated groups. Although the magnitude of these differences was small and unlikely to result in immediate functional consequences, even modest reductions in daily activity may accumulate over time and influence long-term energy balance.

A distinction between free-living PA (i.e., spontaneous or non-obligatory activity performed outside of structured exercise contexts) and structured exercise-related metrics emerged across studies. While free-living PA (e.g., step counts, light-to-moderate intensity PA) tended to be lower in GLP-1 RA-treated groups, structured exercise adherence and prescribed exercise duration were generally comparable between groups. This pattern suggests that GLP-1 RAs may not impair the capacity to engage in planned or supervised exercise, but may be associated with reductions in spontaneous or non-obligatory activity, consistent with adaptations in discretionary energy expenditure [44–46].

This distinction may also help explain discrepancies between objective and subjective PA findings in the literature. Studies relying on self-reported PA have often reported maintained or increased activity levels during GLP-1 RA treatment [47, 48], whereas device-based measures indicate a tendency toward reduced free-living PA. These differences likely reflect both measurement-related bias and variation in pharmacological effects across agents. Self-reported PA is known to overestimate actual activity levels, particularly in individuals with obesity, due to recall bias and social desirability [49–51]. In addition, all studies included in this review evaluated earlier-generation GLP-1 RAs, such as liraglutide and exenatide. Given that newer agents, including semaglutide and tirzepatide, produce greater weight loss and appetite suppression [10, 52, 53], their effects on objectively measured PA may differ from earlier-generation GLP-1 RAs. Ongoing trials incorporating accelerometer-based assessments (e.g., NCT06390501, NCT05173714, NCT04122716) are expected to provide more definitive evidence on these effects.

The exploratory meta-analysis supports these observations. While analyses of three RCTs showed no significant effect, the inclusion of pooled data and non-randomized evidence suggested a modest reduction in PA. This pattern suggests that the observed effects are sensitive to study design and outcome definitions, and that any true effect of GLP-1 RA treatment on PA is likely to be small.

Several physiological and behavioral mechanisms may underlie these findings. GLP-1 RA-induced weight loss is associated with reductions in fat-free mass and resting energy expenditure, which may lead to downstream decreases in total daily energy expenditure [54]. According to the constrained energy expenditure model, such reductions may trigger compensatory decreases in non-essential energy expenditure, including spontaneous physical activity [44, 55]. In addition, GLP-1 RAs may influence central pathways regulating motivation and reward [20]. GLP-1 signaling has been shown to modulate activity in brain regions associated with appetite and reward processing, including the hypothalamus and mesolimbic dopamine system [9, 56–58]. Although direct evidence linking these effects to changes in PA remains limited, modulation of motivational processes may contribute to reduced engagement in free-living activity [59, 60].

GLP-1 RA treatment has also been associated with increases in resting heart rate [10], which may influence perceived exertion during physical activity [24]. While direct evidence linking this effect to reductions in free-living PA is limited, altered physiological responses to exertion may further contribute to behavioral adaptations observed in free-living activity.

From a clinical perspective, these findings suggest that maintaining overall PA during GLP-1 RA treatment may require strategies beyond conventional exercise prescriptions. In particular, interventions should place greater emphasis on sustaining engagement in habitual, free-living PA by targeting the motivational regulation of spontaneous behavior [60], rather than focusing solely on structured exercise adherence.

Building on this perspective, future research should prioritize standardized, objective, and longitudinal assessment of PA to better characterize behavioral responses to GLP-1 RA treatment. Studies involving newer-generation agents are needed to determine whether greater weight loss efficacy is associated with differential effects on free-living activity. Furthermore, a deeper understanding of how pharmacological treatment interacts with motivational and behavioral processes may support the development of more personalized exercise interventions tailored to individual variability in activity responses.

### Limitations

This review has several limitations. First, the number of available studies was small, and sample sizes were limited, reducing statistical power to detect modest effects. Second, all included studies were conducted in Western, high-income settings, which may limit generalizability to populations with different sociocultural and environmental contexts. Third, substantial heterogeneity in PA measurement methods and outcome definitions limited comparability across studies, and intervention durations were relatively short, restricting the assessment of longer-term behavioral adaptations.

In addition, several studies included populations with chronic conditions such as osteoarthritis, cardiovascular disease, and type 2 diabetes, which may independently influence PA levels. Although these populations reflect real-world clinical contexts, it was not possible to fully distinguish the effects of pharmacological treatment from disease-related constraints on activity.

## Conclusions

Current evidence suggests that GLP-1 RA treatment does not significantly alter objectively measured PA in adults with overweight or obesity. However, a consistent tendency toward reduced free-living activity was observed, while structured exercise participation remained largely unchanged. These findings indicate that GLP-1 RAs may be associated with changes in spontaneous activity rather than the capacity to perform prescribed exercise. Although the magnitude of change appears small, such patterns may still have implications for long-term energy balance, underscoring the need for objective monitoring and individualized activity strategies during pharmacological weight management.

## Supplementary information

Supplemental materials

## Data Availability

The primary data analyzed in this study are derived from previously published studies cited in the reference list. The data extraction spreadsheet and meta-analysis code generated during this study are available from the corresponding author upon reasonable request.
